# Community cost-benefit discussions that launched the Camino Verde intervention in Nicaragua

**DOI:** 10.1186/s12889-017-4292-x

**Published:** 2017-05-30

**Authors:** Carlos Hernandez-Alvarez, Jorge Arosteguí, Harold Suazo-Laguna, Rosa Maria Reyes, Josefina Coloma, Eva Harris, Neil Andersson, Robert J. Ledogar

**Affiliations:** 1 CIET, Managua, Nicaragua; 2 Division of Infectious Diseases and Vaccinology, School of Public Health, University of California, Berkeley, CA USA; 3 Centro de Investigación de Enfermedades Tropicales (CIET), Universidad Autónoma de Guerrero, Acapulco, Mexico; 4 Department of Family Medicine, McGill University, Montreal, Canada; 5 CIET International, New York, NY USA

**Keywords:** Community cost-benefit discussions, Community intervention research, Socialising evidence for participatory action

## Abstract

**Background:**

Recent literature on community intervention research stresses system change as a condition for durable impact. This involves highly participatory social processes leading to behavioural change.

**Methods:**

Before launching the intervention in the Nicaraguan arm of Camino Verde, a cluster-randomised controlled trial to show that pesticide-free community mobilisation adds effectiveness to conventional dengue controls, we held structured discussions with leaders of intervention communities on costs of dengue illness and dengue control measures taken by both government and households. These discussions were the first step in an effort at Socialising Evidence for Participatory Action (SEPA), a community mobilisation method used successfully in other contexts. Theoretical grounding came from community psychology and behavioural economics.

**Results:**

The leaders expressed surprise at how large and unexpected an economic burden dengue places on households. They also acknowledged that large investments of household and government resources to combat dengue have not had the expected results. Many were not ready to see community preventive measures as a substitute for chemical controls but all the leaders approved the formation of “brigades” to promote chemical-free household control efforts in their own communities.

**Conclusions:**

Discussions centred on household budget decisions provide a good entry point for researchers to engage with communities, especially when the evidence showed that current expenditures were providing a poor return. People became motivated not only to search for ways to reduce their costs but also to question the current response to the problem in question. This in turn helped create conditions favourable to community mobilisation for change.

**Trial registration:**

ISRCTN27581154.

## Background

### Dengue and the Camino Verde project

In Nicaragua, as in most countries, top-down and pesticide-dependent approaches have failed to curb the spread of dengue. Although efforts are being made to make the struggle to control dengue more participatory [1], until recently the Nicaraguan government strategy for control of the *Aedes Aegypti* mosquito rested on periodic insertion of a packet of an organophosphate, temephos (sold and known popularly under the brand name “Abate”), in water containers of all households, as well as spraying insecticides in urban areas. Resistance of the mosquito to these pesticides is already well documented [2–6] and, given the variable coverage and consistency of temephos use for dengue control, is a growing concern. Nevertheless, the belief persists that mosquito-borne diseases can only be prevented by the application of chemical agents, whether through the action of public health authorities or by purchase and personal or household use of such agents.

In general, interventions that have included education for dengue prevention have not produced the expected improvements in disease transmission. Several trials show an impact of community approaches on vector density but no previous ones have shown impact on dengue illness or serological evidence of infection [7].

CIETinternational has been working with Nicaraguan institutions and communities for more than 20 years gathering evidence to support improvement of government and non-governmental services and solutions to problems as varied as social vulnerability for natural disasters, prevention of HIV, social inclusion linked with tourism, and social control of corruption [8]. The School of Public Health at the University of California at Berkeley (UCB), together with the Sustainable Sciences Institute (www.sustainablesciences.org) has been working with Nicaraguan health authorities since 2004 on dengue virology. In collaboration with the Nicaraguan Health Ministry CIET and UCB conducted a pragmatic randomised controlled cluster trial to test the effect of community mobilisation on dengue incidence in the capital city of Managua. The project had a positive impact on serological evidence of dengue virus infection in children, reported illness at all ages, and all dengue vector control indices [9]. The trial was named *Camino Verde*, Spanish for “green way”.

The method used for this mobilisation was one called Socialisation of Evidence for Participatory Action, or SEPA. Its theoretical background along with examples of its application in other contexts is provided in a companion article [10]. SEPA is a means of partnering with communities to better identify and solve their development challenges based on the participatory search, open circulation, lay interpretation, and collective discussion of local evidence as well as the building of consensus on the choices for action. Unlike most health communication, SEPA does not seek individual behavioural change in and of itself, *but participatory action leading to change* at the national, provincial, district, community, household or individual levels, depending on the issues and the circumstances. Therefore, SEPA is better defined *by its social components and its social and cultural implications* than by individual perceptions and individual cost-weighing.

In this context, risk communication is often used for sharing evidence, but not for prescribing a specific course of action. CIET socialises the evidence for people to negotiate with their own reality, in an informed manner but *in their own terms*, which often implies working out conflicting views and interests in any given society.

In the Nicaraguan context the principal agents of SEPA have been community organizers recruited from their communities who generally work together in teams or “brigades”. Hence the name *brigadistas*.

The evidence they attempt to socialise is of three kinds: entomological, economic, and epidemiological. The entomological evidence concerns the life-cycle of the *Aedes Aegypti* mosquito and the visual evidence of this life-cycle unfolding before householders in their own water receptacles. The economic evidence is the cost data on dengue and dengue control gathered in the baseline survey and from health ministry sources. The epidemiological evidence is that of relative risks and risk differences identified by the analysis of all the data collected. This article focuses on the economic evidence.

### Intervention research and community psychology

The field of community psychology brings the perspectives of social ecology and systems thinking to preventive health interventions in communities [11]. Foster-Fishman defines systems change as “an intentional process designed to alter the status quo by shifting and realigning the form and function of a targeted system” [12]. Pierson and colleagues [13], following Watzlawick [14], distinguish between first-order and second-order change. The former represents the natural progression of a system as it adapts to minor and mostly predictable challenges and events over time. Such change does not alter the elemental structures, functions or culture of the system. In contrast, second-order change intentionally targets the status quo to transform or reframe fundamental system dynamics, structures, resources, rules, norms, and relationships. Parsons argues that “if a system is to make a significant change from its status quo, the changes are likely to come from creative self-organizing rather than from planned change.” ([15], p 407) Hawe and colleagues argue that embracing the systems approach requires us to re-conceptualize the notion of intervention. They propose thinking of interventions as events in systems that either leave a lasting footprint or fade away depending on how well the dynamic properties of the system are harnessed [16].

**Fig. 1:**
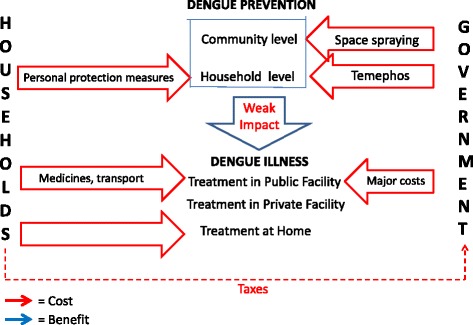
System of dengue-related costs & benefits, Nicaragua, before 2011–2013 Camino Verde trial. *Red arrows* = Costs *Blue arrows* = Benefits

We can conceive of dengue prevention and treatment in Nicaragua as a system of costs that this trial intends to harness and begin to transform in three stages. Figure 1 is a simplified representation of the system as it existed at the beginning of the trial. Red arrows represent costs. Although households are not charged directly for government temephos and fumigation activities, citizens ultimately pay for them with their taxes. They also pay directly for a variety of personal protection devices such as household sprays, fumigating coils and tabs, fans and bednets. Finally, they bear many costs for treatment of dengue cases depending on the seriousness of the illness and the place where treatment occurs.

**Fig. 2:**
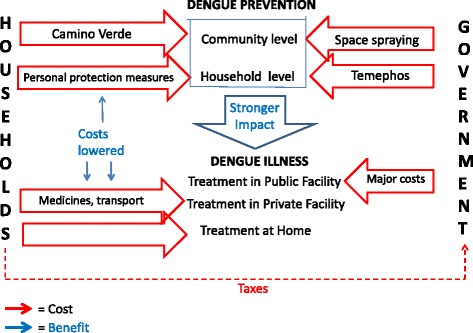
System of dengue-related costs & benefits in intervention communities of Nicaragua at the end of the Camino Verde trial, 2013. *Red arrows* = Costs *Blue arrows* = Benefits

Figure 2 is a simplified representation of the system as it functioned for those in the intervention communities when the trial ended. Government temephos and fumigation efforts continued as before, and taxes continued to support them, in parallel with citizen control of the mosquito breeding environment. While SEPA brigades in Nicaragua were volunteers, the trial provided a small amount of money each month (equivalent to $52 in 2012) to each of the 29 brigades for incidental expenses connected with training, equipment, transport, exhibits, gatherings and peer-monitoring between communities.

**Fig. 3:**
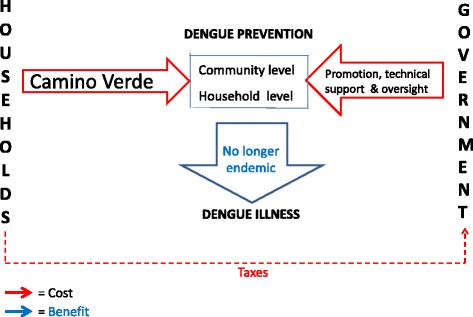
Long term objective of Camino Verde trial for the system of dengue-related costs & benefits in Nicaragua. *Red arrows* = Costs *Blue arrows* = Benefits

Figure 3 is a simplified representation of the system we hope will ultimately result from a wider roll-out of the intervention. Costs to both government and the communities will be significantly reduced while dengue will no longer be endemic in the country.

This third figure does not represent the current situation. The evidence from the follow-up survey needs to be effectively communicated and disseminated and a detailed analysis of the costs and benefits of a wider adoption of the Camino Verde approach will have to be conducted. A likely intermediate step will be the extension of the SEPA intervention to more neighbourhoods in Managua and other parts of Nicaragua. But at some point government, and Nicaraguan society in general, we believe, will realize that the green way is no less effective, less expensive for both government and the communities and more environmentally beneficial than the one it has been pursuing.

According to Hawe and colleagues “the way an intervention comes to seep into or saturate its context becomes a way to view the extent of implementation” ([16], p. 270). The Camino Verde intervention – in the limited number of communities selected for the trial and for the less than 3 years of the trial’s duration -- has “seeped into” personal and household decisions by encouraging people to spend the time to monitor all their water receptacles, clean and cover them. It has seeped into the neighbourhood organization of the intervention by strengthening the bonds between the organization and its membership and demonstrating a way to deal with not only one problem but potentially many others as well. If and when the results of the intervention are recognized and decisions are made to implement it on a wider scale, the change illustrated here will pass, in the language of Pierson and colleagues from a first-order change to a second-order one [13]. Dependence on government action would progressively decrease and conscious collective responsibility for dengue prevention would grow.

Camino Verde was a pragmatic parallel group trial conducted simultaneously in Nicaragua and Mexico and using the SEPA approach in both countries. The SEPA intervention in Nicaragua was many-faceted. The part of the intervention described here was a series of structured discussions with community leaders in Managua held at the start of the intervention on cost-benefit aspects of the current dengue situation. In the companion article on the SEPA approach [10] we explain that unlike some approaches to community engagement in research, such as those of Paulo Freire for example, where the community itself sets the research agenda and may even maintain control over the outcomes of the research, SEPA usually operates within a framework where agendas are set by the providers of research funding. Thus the communities described here did not choose dengue as the subject they most wanted to mobilise around and they were themselves chosen by random assignment. The very nature of the dengue threat dictated many of the actions that needed to be taken to control it. However, within these limitations, communities exercised a large degree of control and displayed considerable interest and ingenuity in the process [10].

These structured discussions follow the Participatory Intervention Model: a framework for conceptualizing and promoting intervention acceptability described by Nastasi et al. [17]. In addition, this project was preceded by a feasibility study in 2004–2007 that identified the use of brigades, composed of community volunteers called *brigadistas*, to act as mobilisers and educators under the authority of the community leadership itself [18].` It was during this feasibility study that the research team became aware of the power that reflections on the costs associated with dengue had as a communication tool to encourage households and communities to consider alternatives to the status quo in mosquito control.

The protocol for the two-country trial explicitly took economic implications of the intervention into account. We intended to document the real cost of preventing dengue using a cost-benefit approach with benefits identified and qualified by communities themselves. We felt that a focus on costs to the household could lead to very different decisions from those imagined by dengue control programmes that rely heavily on temephos and fumigation and remove choice and change the way people estimate their own value in the choices they make [19].

Among both individuals and communities, this intervention aimed not only to control the dengue virus but also to transform **c**onscious knowledge, **a**ttitudes, **s**ubjective norms, intentions to **c**hange prevention behaviour, **a**gency, **d**iscourse and prevention-related **a**ctions. In other settings CIET has found a fairly consistent progression in the process of psychological change and has coined the acronym CASCADA to depict them [20]. We included indicators for measuring change in each of these domains in our baseline survey and repeated them in the impact survey [9].

Our objective in this article is to describe how an intervention aimed at mobilising communities to control the spread of dengue in Nicaragua was set in motion by way of a series of discussions with community leaders in each of the intervention neighbourhoods on costs related to dengue: costs to households for purchase of anti-mosquito products and for dealing with cases of dengue illness as well as costs to the government for treatment of dengue cases. Our objective in the discussions was to obtain the commitment of the community leadership to a collective effort at dengue virus control through non-chemical means.

## Methods

### The baseline survey

A random sample of enumeration areas from the most recent census (2005) provided a panel of 60 clusters stratified by neighbourhood in Managua. These 60 clusters, randomly selected for the intervention, contained 8153 households (approximately 40,693 people). After obtaining consent from local authorities, neighbourhood leaders and the householders themselves, trained and experienced interviewers asked a set of questions from an adult respondent for the household and requested permission for a trained entomological inspector to accompany the household member or members on an entomological assessment of potential *Aedes aegypti* breeding sites on the premises, from which they recorded larvae and pupae counts. ELISA analysis of saliva samples from children aged 3 to 9 years assayed dengue virus-specific IgG. We gathered the saliva samples at the beginning and end of the usual dengue season and classified children as infected if they registered a greater than twofold increase in dengue antibodies. Information gathered included use of temephos, type of water container, other containers near the dwelling, education of household head, involvement in other community matters, number of people living in the household, income sources (salaried or self-employed), perceptions about the social fabric of the neighbourhood (neighbours helping one another, leaders paying attention to one’s opinions, and ability to name organisations and persons that do most to benefit the community).

Specifically in relation to costs, we asked about household expenditure on anti-mosquito products: what products, how frequently they were purchased and how much was spent on each purchase.

Concerning cases of dengue illness, we asked about each member of the household who became sick from dengue in the previous year: where the person was treated (hospital, home, out-patient facility), how many days of work were lost, how many days of school were lost and the amount spent (asking separately about how much was spent on medicines, doctor visit, transport, other).

The baseline surveys and results from the paired saliva samples preceded, and provided stratifying data for, randomisation of the 60 sites into 30 intervention and 30 control clusters.

### Organisation of the discussions

The discussion groups were held in each of the intervention neighbourhoods. Participants were recognised neighbourhood leaders. Low and middle income neighbourhoods in Managua at that time were organized into neighbourhood associations closely allied with the Sandinista government called *Poder Ciudadano* (citizen power).

The discussions were led by facilitators assigned to each intervention neighbourhood. These facilitators were former brigadistas from the intervention neighbourhoods of the feasibility study. They helped to plan the discussions and they conducted them. We drew upon their experience in the process of formulating the discussion questions which they also piloted in other neighbourhoods. As part of the facilitators’ training for the role, an interview guide was prepared with spaces after each question for recording both specific comments by individuals as well as for formulating expressions around which there was agreement, either consensus of the whole group or alternative expression of contrasting views. The principal author of this article synthesized the facilitators’ reports.

To stimulate discussion facilitators made a brief presentation of results from the baseline survey relevant to the specific neighbourhood and gave a short explanation of dengue and its threat to human health. We focused on what families were spending a) to deal with dengue illness when it occurs and b) to try to prevent it by buying pesticide sprays, coils and other devices.

We organized these discussion groups as the first major effort towards building a practice of community dialogue based on evidence. We chose the theme of costs because we believe that cost-benefit decisions by households and communities are central to their efforts to protect themselves. The intention was not to persuade or build consensus on a particular choice or model of action against dengue. With this in mind, we trained facilitators to encourage reflection and to intervene in the discussion themselves only to clarify technical issues and with a clear criterion of respect for all the interpretations that could arise.

In May, June and July of 2011 we held discussion with 30 groups involving an average of 15 people per group; two-thirds were women. Most participants were persons with some responsibility in their community organisation or local politics. In addition to the discussions reported here, these groups also made decisions during the same meetings about the formation of SEPA brigades in their neighbourhoods.

Each participant was given a sheet of paper with four questions, each question accompanied by the relevant evidence. Each question was discussed by the group and the group formulated group responses or commentaries. The questions were:

1. What were the group’s reactions to the costs of caring for people sick with dengue?
2. What comments do group members have about expenditures on chemicals to combat the adult mosquito?
3. What comments do group members have about the appropriateness of spending time searching for larvae and/or pupae?
4. What comments do group members have to the finding that # # out of every 100 homes [the proportion in the specific community] disagree with the statement that the best way to avoid mosquitoes is applying larvicide (Abate) and spraying?

Table 1 presents the *mean* data presented for consideration and discussion. Except for the data on government costs, provided by key informants in the health ministry, all data are from the baseline survey. Each group received and discussed figures specific to its own neighbourhood.

**Table 1:** Data from the baseline survey and government sources discussed with community leaders: Mean results from the 30 intervention

1. Households where someone had dengue in the past year	8.2%
2. Proportion of dengue cases requiring hospitalization	38.0%
3. Expenditure per dengue case	$24.00
4. Expenditure as % of 2010 basic household food basket	9.0%
5. Work days lost by those caring for sick household member	3.87
6. Work days lost by those ill with dengue	8
7. School days lost by those ill with dengue	11
8. Health ministry costs per sick adult	$815
9. Health ministry costs per sick child	$959
10. Proportion of households spending money on products for mosquito prevention/protection	63%
11. Amount spent during month of December 2010 on such products	$5.60
12. Amount spent on such products as a proportion of basic monthly household food basket	2.6%
13. Health ministry expenditure per household per year on vector control (insecticide spraying)	$6.00
14. Proportion of households devoting time to search and removal of mosquito larvae/pupae	32.5%
15. Hours per month spent in this activity	5

Costs in Nicaraguan Cordoba converted here to US$ at the January 5, 2010 rate of C20.85 = US$ 1.00. (Each community received is own specific data)

## Results

### Reactions to the costs of caring for people sick with dengue

Comments generally focused on dengue’s impact on the household economy in two respects, its “high cost” and the fact that it is an “unexpected cost” that none had included in their budgets. It results in “taking away from a planned necessity to cover for the unforeseen event”, and so “the family economy gets unbalanced.”

In many of the groups, people observed that the real costs of such illnesses were always higher than the figure reported, especially where hospitalization was involved, and that the poor “always spend more” or are most affected. Although health care is officially free, health centres often lack the necessary drugs and there are hidden costs for transportation and special feeding needs of the sick.

Group members referred frequently to the unaccounted costs of work days lost due to dengue illness or to caring for the sick, especially among people who are self-employed and/or uninsured. Several groups referred to the risk of losing their jobs due to work absences related to illness.

Discussion of high costs of dengue care both for households and the Ministry of Health led to reflections on the need for, and possibility of, reducing or avoiding these costs and freeing money to meet other health priorities.

### Comments about expenditures on chemicals to combat the adult mosquito?

The discussions tended less toward combating a specific disease like dengue and more toward the more general problem of avoiding “the nuisance of mosquitoes” and even other pests in the house such as mice and roaches.

Almost all groups noted that actual expenses were higher than published official data because these did not include the use of fans (purchase, maintenance and energy expenditure) or bed nets.

For most groups the discussion focused on the ineffectiveness of both household measures and fumigation carried out by the health ministry, with expressions like:

- We spend money every day and the mosquitoes are still there.
- The spraying done by the health ministry and the insecticide (plagatox) purchased by households, only get rid of mosquitoes for a little while; it does not eliminate them and soon they return.
- The Ministry of Health wastes funds because they only spray when there are cases or epidemics, or in the winter and not in every house.
- “We are filling the pockets of the products manufacturers.”
- “We are throwing money into bags with holes in them” by purchasing and using chemicals that do harm to both our health and the environment.

These observations led most groups to the conclusion that the costs associated with these products should be reduced or even eliminated. A few groups, on the other hand, concluded that the health ministry should reinforce its fumigation programme (more often, throughout the year and in all households).

### Comments about the appropriateness of spending time searching for larvae and/or pupae

Answers to this question were the most diverse. Most groups agreed that very few households take time to find and remove larvae/pupae. Typical statements were:

- “People don't eliminate larvae because they are not aware of them or don't know what they look like. Nobody has taught them.”
- “People don't take the time because they have to work.”
- “Households have always relied on *Abate* to solve the problem.”
- “There are houses in which people are just filthy or expect other people to do the cleaning.”
- “The problem is that the health ministry doesn't apply *Abate* frequently enough, they don't apply it to all potential breeding sites or they are using *Abate* that has passed its expiration date.”
- “There are houses that won't open their doors to fumigation or clean-up brigades.”

While most of the groups concluded the need to educate people to clean up the water receptacles in their homes, several of them also expressed the need to combine this with the proper application of *temephos*.

Several groups made reference to the poor performance of institutions such as ENACAL (water supply) or the Municipality (garbage collection, cleaning of gutters and drains) that favour the breeding of larvae in homes or public places, and called for their increased participation in the fight against dengue.

### Comments on the finding that # # out of every 100 homes [the proportion in the specific community] disagree with the statement that the best way to avoid mosquitoes is applying larvicide (Abate) and spraying

Five groups concluded that “larvicides and spray are the best way” and two were ambivalent, but they all also expressed the need to complement this with cleanings from within the household. Other groups concluded that the best way is to eliminate breeding sites in each house. The most common expressions associated with these positions were:

- “XX% [of the neighbourhood] does not want to make progress or prevent disease, but we must respect them”
- “They are insurgents because they distrust the *Abate*.”
- “Those are the Liberals [political party opposed to the party in power] and are the ones who do not like opening their door to the government teams when they are applying *Abate.*”
- “We have to complement the larvicides and spraying with household cleaning for best results.”
- “Even with fumigation and *Abate* there is much household expense, there are mosquitoes and there are cases of dengue.”
- “They have no knowledge of how to prevent and do not know the side effects of chemicals.”
- “Chemicals are not effective. Relying on *Abate* and fumigation is a very comfortable stance.”

Whatever choices they favoured, most of the groups mentioned the need to lower household and health ministry expenditures to devote them to other priorities. All groups indicated, in one way or another, the need to distribute to all households in the neighbourhood this information about the threat of the dengue-bearing mosquito as well as the costs of dengue and its control.

Many were not ready to see community preventive measures as a substitute for chemical controls but all the leaders approved the formation of “brigades” to promote chemical-free household control efforts in their own communities (because of problems that arose subsequently, two of the 30 communities eventually withdrew from the trial.)

The government’s vector control programme, including delivery of temephos and fumigation activities continued throughout the trial while all activities of the brigades were chemical-free.

## Discussion

It was clear in all the groups that the evidence presented had the power both to encourage dialogue and to elicit diverse interpretations. The realisation of how large an economic burden dengue places on households and the awareness of the unsatisfactory results from large investments of household and government resources to combat dengue opened their minds to the possibility that the community itself could act to both lower household and public expenditure and have a significant impact on the dengue problem.

### Lessons for adapting the intervention

**Fig. 4:**
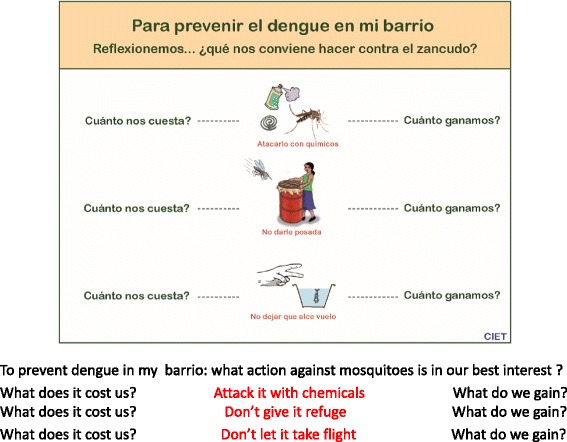
Laminated card carried by Managua Brigadistas

The discussions provided the research team with several important lessons for fine-tuning the intervention to increase the likelihood of its success:

- Although we had earlier contacts with the leadership of these Managua neighbourhoods, these discussions gave us further insight into the particularities of each neighbourhood and the unique dynamics of the leaders’ relations with the people and among themselves. They also helped us to understand the structures that underpinned community organization in every district, and even of the subdivisions in some of the neighbourhoods. Knowledge and understanding of different forms of communication, management styles, internal conflicts and how they played out helped the field teams promote the project in each neighbourhood and assure its continuity in ways particular to that community. Some might fear that such an adaptive approach could compromise the fidelity of the intervention to its design, but Hawe and colleagues have shown that the conventional view of fidelity does not apply to the type of intervention research undertaken here. It is not the form of the intervention that needs to be standardized across sites but rather its function [21]. SEPA is neither a recipe nor a model to be adjusted according to particular circumstances. It is an approach that serves a common function of assisting communities – always with respect for their knowledge and customs -- to identify ways of reaching common objectives [9].
- Dengue had not always been a top priority for the leadership of all these communities. Health was only one of their concerns and they had generally relied on the municipal and national health authorities to deal with public health problems. This and the tendency of the leaders to discuss prevention measures not in terms of dengue alone but of all mosquito-borne nuisances suggest that the intervention’s impact should be evaluated not in terms of cost-effectiveness but from a cost-benefit perspective. We expected increased social capital, understood as an increase in such attributes of community life as trust, reciprocity, collective action, and participation, to be an important benefit of this intervention. Identifying the added effectiveness of informed community mobilisation on social capital was a specific objective of the trial [19].
- Community leaders appeared quite aware that large investments of household and government resources to combat dengue have not produced the expected results. They placed much of the blame for this on inefficiencies in the system that delivers the chemical control substances. They seemed only indirectly aware of possible chemical resistance in the Aedes mosquito. This is an economic cost as it seriously inhibits temephos’s ability to do the job for which it was purchased and applied. We were able to reinforce this aspect of the system in subsequent SEPA discussions.
- It was clearly unwise to advocate a rapid phase-out of the government’s temephos and fumigation activities. Several groups concluded that the best way to avoid dengue is by way of larvicides (temephos) and fumigation. But even these groups decided to form their own SEPA brigades, either because they considered that the official strategy should be reinforced by changes in the household or they wanted to judge for themselves which strategy would have the best results. Moving to a system relying entirely on the Camino Verde approach would require more time and continuing strong community commitment.
- The brigades used the cost-benefit considerations discussed with the leadership at the beginning of the intervention during subsequent house-to-house visits in each of the neighbourhoods. Figure 4 shows one side of a laminated card carried by each brigadista during household visits that asks three questions about the costs and benefits:

- What does it cost to purchase anti-mosquito products and what benefit to we get from it?
- What does it cost us to cover water barrels so as to prevent mosquitoes getting into them to lay their eggs, and what benefit do we get from doing that?
- What does it cost us to remove mosquito larvae and pupae from water receptacles and what benefit do we get from doing that?

### Social psychology and behavioural economics

The community leaders’ reactions to the cost data revealed in these discussions display several aspects of what behavioural economists call “bounded rationality”. When calculating expected utility many human decisions are not fully thought through and can be constrained by the limits of time and cognitive capability [22, 23].

People’s reliance on government to solve the mosquito problem without taking measures available to them to control mosquito breeding in their own households is an example of bounded rationality. Another example is the evidence from these community discussions that, even though dengue was affecting one in 12 households, people generally did not foresee it affecting them and did not allow for the concomitant costs in their economic calculations. As noted at the beginning of the Results section, the costs associated with dengue were unexpected costs; none had included such costs in their budgets. Behavioural economists have shown that people often underestimate their chances of developing serious illness [24].

One way to change this tendency to underestimate the likelihood of illness is by seeking to change the social norms associated with it. Neo-classical economic theory assumes we independently know what we want and that our preferences are fixed. But there is a considerable body of social psychological research suggesting that the existence and strength of social norms influence people’s actions in important ways [25].

Because the SEPA strategy addresses both individual choice and its community context, we believe it has the power to change both norms and behaviour. As long ago as 1951 Lewin argued that the process of ‘unfreezing’ existing behaviour patterns needs to take place in a group environment and to involve open and supportive communication among those involved in negotiating the change [26]. More recently, with respect to environmental issues, Kaplan has maintained that providing people with opportunities for understanding, exploration and participation engages powerful motivations for competence, making oneself useful, making a difference and forging a better life [27]. And Jackson has argued that changing behaviour cannot be conceived as the processes of encouraging change at the individual level; rather pro-environmental behavioural change has to be a social process [25].

We have other evidence that systematic, evidence-informed, discussion by community leaders can have a significant influence on community behaviour. In a randomised cluster controlled trial in Pakistan, Andersson and colleagues demonstrated the effect that informed discussion of vaccination costs and benefits could have on vaccination uptake [28].

The discussions reported here were just one part, of a two-year process of education and dialogue with residents and leaders that led to greater confidence in the green option [9].

## Conclusions

A systems approach to community intervention research helps researchers identify and target those parts of systems that need changing and the relationships that are crucial to achieving change. Reflecting on our trial in terms of a system of costs and benefits and organising a series of structured discussions about these costs and benefits with community leaders enabled us to see several aspects of the intervention that needed further refining.

Discussions that are centred around household budget decisions provide a good entry point for researchers to engage with communities, especially when the evidence shows that current expenditures are providing a poor return. People become motivated not only to search for ways to reduce their costs but also to question the current response to the problem. This in turn helps create conditions favourable to community mobilisation for change.

## Abbreviations

CASCADA: Conscious knowledge, change in Attitude, positive deviations from negative Subjective norms, intention to Change, perception of Agency to change, Discussion of possible action, and Action
ELISA: Enzyme-linked immunosorbent assay
ENACAL: *Empresa nacional de aguas y alcantarillados* (National water and sewerage company)
SEPA: Socialisation of Evidence for Participatory Action
UCB: University of California at Berkeley
